# Flavonoids as Putative Inducers of the Transcription Factors Nrf2, FoxO, and PPAR*γ*

**DOI:** 10.1155/2017/4397340

**Published:** 2017-07-06

**Authors:** Kathrin Pallauf, Nils Duckstein, Mario Hasler, Lars-Oliver Klotz, Gerald Rimbach

**Affiliations:** ^1^Institute of Human Nutrition and Food Science, Christian-Albrechts-University, Kiel, Germany; ^2^Lehrfach Variationsstatistik, Christian-Albrechts-University, Kiel, Germany; ^3^Department of Nutrigenomics, Institute of Nutrition, Friedrich Schiller University, Jena, Germany

## Abstract

Dietary flavonoids have been shown to extend the lifespan of some model organisms and may delay the onset of chronic ageing-related diseases. Mechanistically, the effects could be explained by the compounds scavenging free radicals or modulating signalling pathways. Transcription factors Nrf2, FoxO, and PPAR*γ* possibly affect ageing by regulating stress response, adipogenesis, and insulin sensitivity. Using Hek-293 cells transfected with luciferase reporter constructs, we tested the potency of flavonoids from different subclasses (flavonols, flavones, flavanols, and isoflavones) to activate these transcription factors. Under cell-free conditions (ABTS and FRAP assays), we tested their free radical scavenging activities and used *α*-tocopherol and ascorbic acid as positive controls. Most of the tested flavonoids, but not the antioxidant vitamins, stimulated Nrf2-, FoxO-, and PPAR*γ*-dependent promoter activities. Flavonoids activating Nrf2 also tended to induce a FoxO and PPAR*γ* response. Interestingly, activation patterns of cellular stress response by flavonoids were not mirrored by their activities in ABTS and FRAP assays, which depended mostly on hydroxylation in the flavonoid B ring and, in some cases, extended that of the vitamins. In conclusion, the free radical scavenging properties of flavonoids do not predict whether these molecules can stimulate a cellular response linked to activation of longevity-associated transcription factors.

## 1. Introduction

Flavonoid consumption via diet may benefit cardiovascular health in humans [1, 2], and in some cases, flavonoid supplementation prolonged lifespan of lower model organisms such as flies and worms [3]. Since many flavonoids are known to act as free radical scavengers, putative health benefits were partly attributed to their direct antioxidant capacity. However, it has become apparent that flavonoids modulate signalling processes in cultured cells and possibly also in vivo [4]. By inducing redox-sensitive transcription factors such as nuclear factor (erythroid-derived 2)-like 2 (Nrf2) or forkhead box O (FoxOs), these polyphenols could prevent oxidative damage. While Nrf2 controls genes encoding proteins that counteract oxidative stress and detoxify xenobiotics [5, 6], it may also regulate genes involved in cell survival, metabolism, and adipocyte differentiation [7]. There are four FoxOs (FoxO1, FoxO3, FoxO4, and FoxO6) in humans which all bind to the same consensus sequence. FoxOs are important for cellular homeostasis and can induce cell survival or cell death [8]. They appear to be central for stress response [9] and may affect longevity [10]. Another transcription factor that might be modulated by flavonoids is peroxisome proliferator-activated receptor gamma (PPAR*γ*). It is crucial for adipocyte differentiation [11], and regulation of PPAR*γ* by polyphenols may ameliorate diabetes [12]. Interestingly, FoxO1 was shown to repress adipocyte differentiation via PPAR*γ* [13]. In vitro experiments have demonstrated the activation of Nrf2, PPAR*γ*, and FoxOs by flavonoids [11, 14-16]. In various cell models, the flavonol quercetin induced all three transcription factors [16-18]. Moreover, in vivo data points to the notion that flavonols and flavones may exert their health-benefitting effects via these transcription factors [19-21].

**Figure 1:**
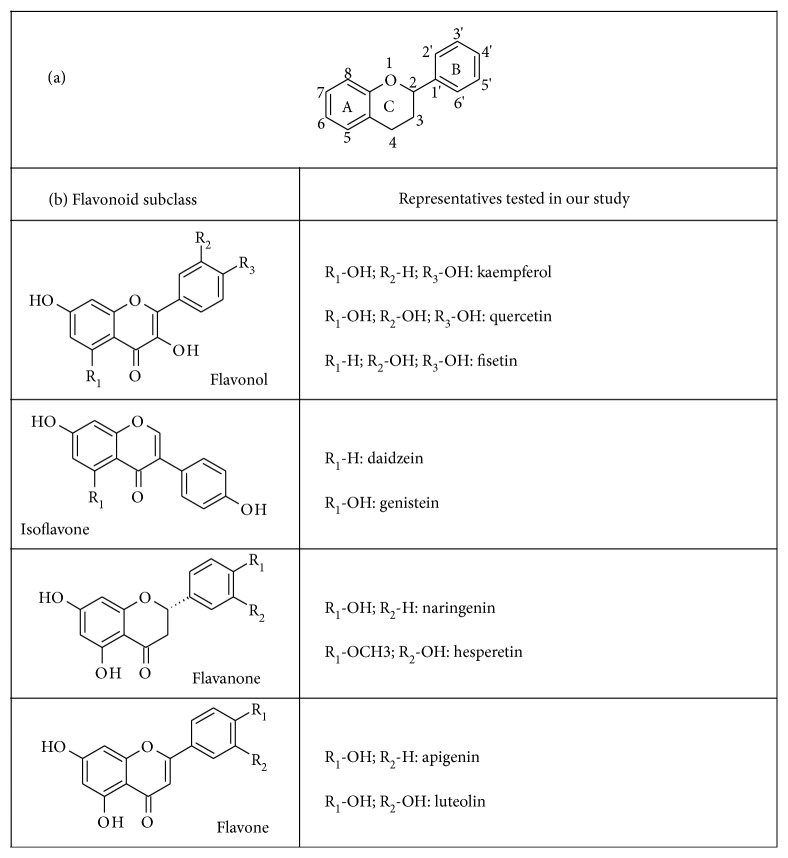
(a) Flavan structure (b) flavonoids used in this study.

To study and compare the activation of Nrf2, FoxO, and PPAR*γ* by flavonoids belonging to different flavonoid subclasses, we tested various flavonols, flavones, isoflavones, and flavanones (Figure 1) in cultured Hek-293 cells transfected with suitable reporter gene constructs. We measured the activity of the flavonols kaempferol and quercetin, which have two hydroxy groups at the A ring and one or two hydroxy groups, respectively, at the B ring. Furthermore, we tested fisetin, which differs from the aforementioned flavonols in having only one hydroxy group at the A ring, as well as apigenin and luteolin, which are the flavone counterparts to kaempferol and quercetin, respectively. From the flavanone subclass of flavonoids, we picked naringenin with a para hydroxy group at the B ring and hesperetin with a meta hydroxy group and a para methoxy group at the B ring. The isoflavones we used, genistein and daidzein, differ in their number of hydroxy groups at the A ring (genistein has two and daidzein has one) while both have one para hydroxy group at the B ring.

**Figure 2:**
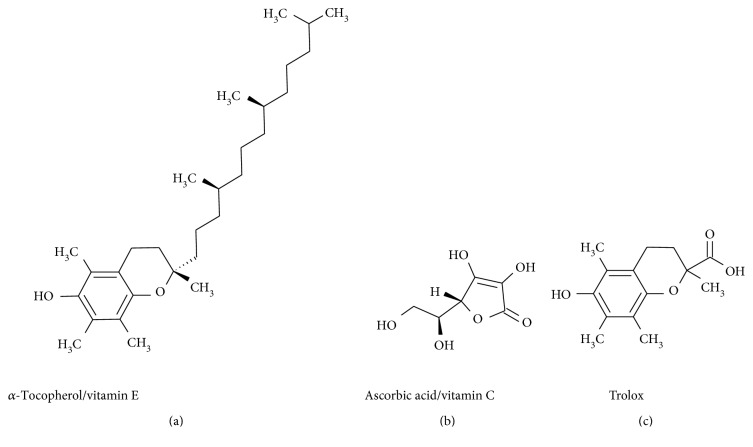
Chemical structure of ascorbic acid (b), *α*-tocopherol (a), and trolox (c).

In addition to the activation of the transcription factors, we assessed flavonoid antioxidant activity by analysing their ability to reduce the organic radical derived from 2,2′-azino-bis(3-ethylbenzothiazoline-6-sulfonic acid) (ABTS assay) or a Fe(III) complex (FRAP assay) under cell-free conditions and using the water-soluble vitamin E orthologue trolox (Figure 2) as a control. For both the analyses of the transcription factor activation and the antioxidant activity, we used the antioxidants ascorbic acid and *α*-tocopherol as comparisons (Figure 2).

## 2. Materials and Methods

### 2.1. Cell Culture

Hek-293 cells (German collection of microorganisms and cell cultures, Braunschweig, Germany) were maintained in Dulbecco's modified Eagle's medium (DMEM) containing 4.5 g/L glucose, 4 mM L-glutamine, 1 mM sodium pyruvate (PAN Biotech, Aidenbach, Germany), 10% fetal calf serum (Gibco, via Thermo Fisher, Darmstadt, Germany), 100 U/mL penicillin, and 100 *μ*g/mL streptomycin (PAN Biotech, Aidenbach, Germany). Cells were grown in 5% CO_2_ at 37°C under a humidified atmosphere. All cell-culture plasticware was purchased from Sarstedt (Nuembrecht, Germany). For all cell culture assays, vehicle controls were performed and did not affect any of the parameters measured.

### 2.2. Transient Transfection and Luciferase Reporter Gene Assay

Hek-293 cells were grown to 60% confluence in 24-well plates for 24 h. The cells were transiently transfected with a firefly luciferase expression vector or expression system (pGl3-NQO1-ARE, pGl3-FHRE∆XRE, or pUAS(4^∗^)-TK-Luc together with pM1-hPPAR*γ*-LBD). For normalization, a renilla-expressing plasmid was cotransfected. Transfection was performed using JetPei transfection reagent (Polyplus transfection, Illkirch Cedex, France) according to the manufacturer's instructions. Following 24 h of transfection, cells were incubated with the test compounds for 24 h in 10% serum-containing medium. Subsequently, cells were lysed and luciferase activity was measured using the dual-luciferase reporter gene assay system (Promega, Mannheim, Germany) in a Tecan Infinite 200 microplate reader (Tecan Group Ltd., Crailsheim, Germany) according to the manufacturer's protocol. A minimum of three independent experiments was performed.

### 2.3. Plasmids

pGl3-NQO1-ARE and pGl3-FHRE∆XRE have a pGl3 backbone (Promega, Mannheim, Germany) which contains a multiple cloning site and a SV40 promoter upstream of a firefly luciferase gene that functions as reporter gene. Fragments containing the binding sites for Nrf2 (pGl3-NQO1-ARE) and FoxO (pGl3-FHRE∆XRE) were inserted using the multiple cloning sites to precede the SV40 promoter, thereby functioning as enhancers for luciferase expression. pGl3-NQO1-ARE had a 32 bp oligonucleotide derived from rat NAD(P)H:quinone reductase mRNA that contains an ARE-motif inserted into the pGl3 backbone [22]. pGL3-FHRE∆XRE was constructed from addgene plasmid 1789 [8] by removing a xenobiotic response element (XRE) [23].

For measuring PPAR*γ* activation, we used Gal4-directed gene transcription which is widely employed to measure gene expression [24]. The Gal4 (yeast transcription activator protein) fused to the PPAR*γ* ligand-binding domain (LBD) and a firefly luciferase construct under the control of UAS (upstream activating sequence) were used. Upon ligand binding to PPAR*γ*-LBD, the fused Gal4 can bind to UAS and thereby induce luciferase expression. In our model, overexpression of PPAR*γ* was advantageous because PPAR*γ* expression in cells other than adipocytes is rather low [25]. pM1-hPPAR*γ*-LBD and the pUAS(4^∗^)-TK-Luc vector were a kind gift from Karsten Kristiansen (Department of Biochemistry and Molecular Biology, University of Southern Denmark, Odense, Denmark) [26, 27].

The normalization vector phRL-TK was from Promega, Mannheim, Germany.

### 2.4. Flavonoids and Vitamins

Quercetin, fisetin, hesperetin, naringenin, and *α*-tocopherol were from Sigma-Aldrich, Darmstadt, Germany; daidzein and kaempferol from Biorbyt, Cambridge, UK; genistein and ascorbic acid from Carl Roth, Karlsruhe, Germany; luteolin from Cayman Chemicals, Ann Arbor, Michigan, US; apigenin from Selleck Chemicals, Munich, Germany; and trolox from Fluka via Sigma-Aldrich. Ascorbic acid and trolox were dissolved in water, *α*-tocopherol in ethanol (Normapur®, VWR, Darmstadt, Germany), and the flavonoids in DMSO (Carl Roth) at 100 mmol/L for preparing stock solutions.

### 2.5. Neutral Red Assay

Cytotoxicity was determined via the neutral red assay [28, 29]. Hek-293 cells were seeded in 24-well plates (Fisher Scientific, Schwerte, Germany) at a density of 120,000 cells/well, precultured for 24 h, and treated with the flavonoids, ascorbic acid, or *α*-tocopherol at concentrations ranging from 1 to 200 *μ*M for 24 h in 10% serum-containing DMEM. Then, the culture medium containing the test substances was replaced with fresh serum-containing medium including 50 *μg*/mL of neutral red (Carl Roth). After incubation for 3 h, the medium was removed and the cells were extracted using a solution comprising 50 : 49 : 1 (*v*/*v*/*v*) ethanol, water, and glacial acetic acid (Carl Roth). The absorbance was measured in a plate reader (Labsystems, Helsinki, Finland) at 540 nm. Based on these toxicity tests, we chose the highest nontoxic concentration of the most toxic compound for the luciferase assays (20 *μ*M for the flavonoids).

### 2.6. Antioxidant Capacity Assays

#### 2.6.1. ABTS Assay

The ABTS assay measures how well a test compound can reduce 2,2′-azino-bis(3-ethylbenzothiazoline-6-sulfonic acid) (ABTS) radicals which are formed by oxidation of ABTS with potassium persulfate. Antioxidants can scavenge this blue green radical and thereby decolour the test solution which can be measured photometrically [30].

A 7 mM ABTS and 2.45 mM potassium persulfate (both Sigma-Aldrich, Darmstadt, Germany) solution was diluted with water to give an absorbance of 0.7 at 690 nm. Following the addition of the test compound (or the vehicle control) to yield a total volume of 310 *μ*L, and 6 min of incubation at room temperature, absorbance at 690 nm was measured in a Tecan Infinite 200 microplate reader (Tecan Group Ltd., Crailsheim, Germany). The results were plotted as the differences in absorbance relative to the vehicle control against the concentrations of the tested compound (the larger the difference, i.e., the greater the loss of absorbance, the more extensive is the reduction of the ABTS radical).

#### 2.6.2. FRAP Assay

The ferric-reducing ability of plasma (FRAP) assay measures how well a test compound can reduce ferric (i.e., iron-III) to ferrous (i.e., iron-II). Ferric ion (iron-III) is turned into ferrous ion (iron-II) at low pH upon addition of a reducing agent. The formation of ferrous iron can be measured photometrically in a 2,4,6-tris(2-pyridyl)-s-triazine (TPTZ) solution since iron (II) forms a coloured complex with TPTZ [31].

Following addition of the to-be-tested compound (or the vehicle control) to an iron (III) chloride solution (1.7 mM) with TPTZ (1.67 mM) in acetate-buffered solution (228 mM) at pH 3.6 and 15 minutes of incubation, absorbance at 620 nm was measured. The absorbances resulting from ferrous ion/TPTZ complex formation were plotted against the concentrations of the tested compound.

FRAP and ABTS measurements were carried out in a Tecan Infinite 200 microplate reader (Tecan Group Ltd., Crailsheim, Germany). The final concentrations of the flavonoids and vitamins measured were 645, 323, 161, 65, 32, and 0 (solvent control) nM. In order to calculate the gradient relative to trolox, linear regressions were carried out and the gradient from the plotted flavonoid/vitamin curve was divided by the trolox gradient.

All experiments were carried out a minimum of three times (different days).

### 2.7. Statistics

The statistical software R [32] was used to evaluate the data. Data evaluation started with the definition of an appropriate mixed model [33, 34]. The data was assumed to be approximately normally distributed. These assumptions are based on a graphical residual analysis. For the reporter gene assays, the treatment was regarded as a fixed factor and the day as a random factor. Based on this model, a pseudo *R*^2^ was calculated [35] and an analysis of variances (ANOVA) was conducted, followed by multiple contrast tests (Dunnet) [36] to compare the firefly/renilla ratios.

For the FRAP and ABTS assays, the treatment and the concentration were regarded as fixed factors and the day as random factor.

Based on this model, an analysis of variances (ANOVA) was conducted, followed by multiple contrast tests (Dunnet) [36] to compare the ∆ absorbance at 690 nm for the ABTS assay and the absorbance at 620 nm for the FRAP assay.

Correlations and *p* values were calculated using “rcorr” type “Pearson” (ABTS, FRAP, and reporter gene assays with each other) or “Spearman” (number of hydroxy groups with assay outcome) from the package “Hmisc.”

## 3. Results and Discussion

### 3.1. Flavonoids but Not Antioxidative Vitamins Activate Longevity-Associated Transcription Factors in Hek-293 Cells

**Figure 3:**
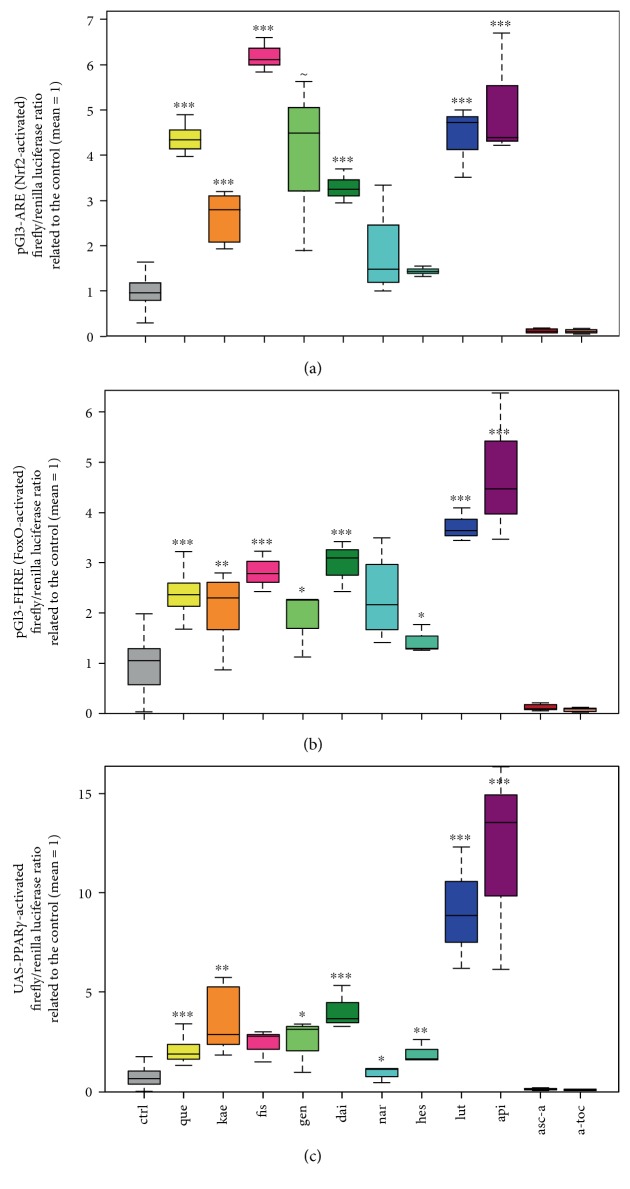
Hek-293 cells were transfected with firefly luciferase constructs controlled by elements responding to Nrf2 (a), FoxO (b), or PPAR*γ* (c) activation. Constitutively expressed renilla luciferase was cotransfected to obtain firefly/renilla ratios. The vehicle for the tested flavonoid or vitamin and quercetin as a positive control were included in every experiment. In order to show all experiments in one plot, the firefly/renilla luciferase ratios were normalised to the difference between the control and quercetin and the mean of the control was set to be 1. Concentrations of flavonoids and vitamins were 20 *μ*M and 100 *μ*M, respectively. ctrl: vehicle control; que: quercetin; kae: kaempferol; fis: fisetin; gen: genistein; dai: daidzein; nar: naringenin; hes: hesperetin; lut: luteolin; api: apigenin; asc-a: ascorbic acid; a-toc: *α*-tocopherol; ARE: antioxidant response element; FHRE: forkhead responsive element; PPAR: peroxisome proliferator-activated receptor; UAS: upstream activating sequence. ~*p* < 0.1 compared to the control, ^∗^*p* < 0.05 compared to the control, ^∗∗^*p* < 0.01 compared to the control, and ^∗∗∗^*p* < 0.005 compared to the control. For the statistics, the non-normalised firefly/renilla ratios were used. A minimum of three independent experiments was performed.

To analyse Nrf2 activation, we measured antioxidant response element- (ARE-) driven firefly luciferase expression (pGl3-ARE) in Hek-293 cells cotransfected with the plasmid phRL-TK constitutively expressing renilla luciferase (Figure 3(a)). ARE is a binding site in the promotor region of Nrf2 target genes [6]. Similarly, FoxO transcription factors bind the forkhead responsive element (FHRE) [9] and we used a pGl3-FHRE firefly luciferase construct to measure FoxO activation (Figure 3(b)). To measure PPAR*γ* activation, we used the Gal4 (yeast transcription activator protein) bound to the PPAR*γ*-LBD and a firefly luciferase construct that was under the control of UAS (Figure 3(c)).

The flavonol quercetin gave positive results in all our experiments, and this is in accordance with various reports on the induction of redox-sensitive transcription factors [16, 29, 30]. Therefore, we included quercetin and the vehicle control as positive and negative control, respectively, in every set of luciferase assays.

The flavones luteolin and apigenin, which differ from quercetin by lacking the hydroxyl group in the C ring (luteolin) as well as the meta hydroxylation in the B ring (apigenin) (Figure 1), appeared to be the most active flavonoids tested. Interestingly, the flavonol fisetin, which differs from quercetin in lacking one hydroxy group in the A ring, also appeared very potent in the ARE assay but did not reach significance in the PPAR*γ* assay.

In contrast, luteolin and apigenin were highly active in all three reporter gene assays. Remarkably, most of the compounds tested showed similar potencies to activate all three transcription factor-responsive assays (Figure 3).

Kaempferol, which is a flavonol-like quercetin but with one hydroxy group less in the B ring (Figure 1, Table 1), also showed induction in all three assays. While kaempferol appeared a weaker inducer than quercetin in the Nrf2- and FoxO-responsive assays, it seemed stronger than quercetin in the PPAR*γ*-responsive assay (Figure 3).

The isoflavonoids daidzein and genistein seem to be moderate and weak inducers, respectively, in all three assays. These two isoflavonoids differ from each other in having one (daidzein) or two (genistein) hydroxy groups in the A ring. The flavanone hesperetin appears to be a weak inducer of FoxO- and PPAR*γ*-driven reporters but showed no significant effect on Nrf2. Naringenin, which, compared to hesperetin, has a demethylated para hydroxy group and no meta hydroxy group in the B ring, seemed slightly less active than hesperetin and only showed significant activation in the PPAR*γ* assay (Figures 1 and 3).

Consistent with the data from lifespan studies showing that ascorbic acid and *α*-tocopherol do not extend lifespan in model organisms [37, 38], neither vitamin C nor vitamin E induced PPAR*γ*, FoxO, or Nrf2-driven luciferase expression (Figure 3).

Wang et al., Bumke-Vogt et al., Lee et al., Saw et al., and Paredes-Gonzalez et al. [39–43] reported that flavonols and flavones were relatively potent inducers of the longevity-associated transcription factors Nrf2, FoxO, and PPAR*γ*. Moreover, it was reported that fisetin stimulated Nrf2 signalling, ERK/MAPK signalling, and kinases involved in cell cycle regulation in vitro [44]. Of interest, flavonoids were shown to influence various cyclin-dependent kinases [45], mitogen-activated protein kinases (MAPK), protein kinase Akt, and FoxO signalling [46] and to counteract inflammation [47-49]. In the roundworm, *Caenorhabditis elegans*, quercetin, kaempferol, fisetin, and naringenin supplementation induced nuclear translocation of the *C. elegans* FoxO orthologue [50, 51]. While FoxO3 single nucleotide polymorphisms have been associated with longevity [52], it remains unclear whether flavonoids exert their lifespan-extending effects observed in model organisms via FoxO. On the one hand, only few flavonoids depend on worm FoxO to exert lifespan extension [53]. On the other hand, in a transgenic mouse model for prostate cancer (TRAMP mice), apigenin inhibited cancer, in part, via FoxO [19].

These effects on cellular signalling pathways may contribute to the health-benefitting findings from epidemiological studies [54]. However, when evaluating the effect of dietary flavonoids, it should be kept in mind that different flavonoids, besides affecting numerous signalling pathways, [55] may act synergistically or antagonistically. Furthermore, when comparing data from in vivo studies with our results, it is important to keep in mind that we were working with flavonoid aglycons at supraphysiological concentrations. Most flavonoids in vegetables and fruits are glycosylated and they may be transported by the SGLT1 or hydrolysed and absorbed as aglycons [56]. Yet, once absorbed, they are readily metabolised by methylation, glucuronidation, and sulfation [56], which is why aglycon concentration in tissues or plasma is very low.

Our data obtained does not indicate obvious structure-activity relationships for the induction of Nrf2, FoxO, or PPAR*γ* by flavonoids. The flavone to flavonol counterparts apigenin to kaempferol and luteolin to quercetin have one or two hydroxy groups, respectively, in the B ring and were active in all luciferase assays. While in the Nrf2-induced luciferase assay, quercetin showed more activity than kaempferol; the flavone with two hydroxy groups in the B ring (luteolin) did not show stronger induction than apigenin (Figure 1). Consistently, we did not find significant correlations between the number of hydroxy groups and activities in the reporter gene assays (Spearman correlation Table 2). However, the differences in luciferase assay activation by flavonoids could also be due to different degrees of protein binding, stability, or flavonoid concentrations in the cell [57].

### 3.2. Patterns of Flavonoid-Induced Activation Are Similar for Nrf2-, FoxO-, and PPAR*γ*-Dependent Reporters

Interestingly, the different flavonoids showed similar capacities to induce all three transcription factors. This can be seen by looking at the box plots (Figure 3) and the correlation coefficients ARE versus FHRE: *R* = 0.85; PPAR*γ* versus ARE: *R* = 0.64; and PPAR*γ* versus FHRE: *R* = 0.67 (Table 1). Since Nrf2 and FoxOs are redox-sensitive transcription factors, it seemed somewhat plausible that their responsive elements ARE and FHRE were activated by similar stimuli. However, ARE and FHRE activation also correlated with PPAR*γ* activation.

PPAR*γ* controls adipocyte differentiation and is activated by endogenous agonists such as fatty acids [58] and xenobiotics such as rosiglitazone [59]. The thiazolidinedione was used as an antidiabetic drug until it became evident that its use was associated with increased risk of myocardial infarction [60]. Examples of flavonoids that were shown to be agonists or partial agonists of PPAR*γ* are the flavonols kaempferol and quercetin [61], the flavones luteolin [62] and apigenin [63], and the isoflavones daidzein [64] and genistein [65]. It has been hypothesized that plant-derived PPAR*γ* modulators may be able to improve insulin sensitivity without detrimental side effects. Of interest, dietary supplementation of high-fat-fed mice with luteolin ameliorated insulin resistance [62].

Furthermore, PPAR*γ* may participate in antioxidant response since it shares target genes such as those coding for heme oxygenase 1 and catalase with Nrf2 [13] and was shown to be regulated by Nrf2 and FoxO [66-70]. However, in our model, we overexpressed the PPAR*γ*-LBD fused to Gal4 which would induce luciferase expression via activation of the UAS. Thus, flavonoids stimulating this assay would be expected to fit into the PPAR*γ*-LBD (functioning as agonists or partial agonists) rather than to induce transcription or translational modifications of PPAR*γ* or its cofactors [71]. Even so, putative positive feedback loops in between FoxO or Nrf2 and PPAR*γ*-signalling [13] could have contributed to our experimental outcome. Consistent with an interaction between Nrf2 and PPAR*γ* to combat oxidative stress, genistein-mediated protection from stress-induced cell injury depended on both transcription factors [72]. Moreover, flavonoids seem to activate upstream signalling molecules such as PKC which phosphorylates Nrf2 thus enabling its further activation [41, 73]. Stimulation of molecular targets upstream of Nrf2, FoxO, and PPAR*γ* could in part explain why the flavonoids we tested activated all three transcription factors to a similar extent.

### 3.3. Reporter Gene Activation Patterns Elicited by Flavonoids Do Not Correlate with Their Antioxidant Activities

**Figure 4:**
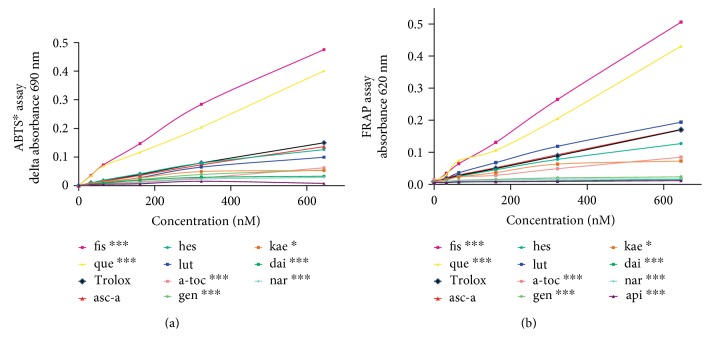
Graph showing the antioxidant capacity of the flavonoids and vitamins tested compared to trolox in assays using the 2,2′-azino-bis(3-ethylbenzothiazoline-6-sulfonic acid) (ABTS) radical (a) and the ferric-reducing ability of plasma (FRAP) (b). (a) The reduction of the coloured ABTS radical is plotted as the difference in absorbance to the vehicle control against the concentration of the compound that was added to the reaction. Absorbance was measured at 690 nm after 6 minutes. (b) The absorbance of a solution containing a known concentration of ferrous ions is plotted against the flavonoid or vitamin concentration. Absorbance was measured at 620 nm after 15 minutes incubation. The legends show the flavonoid with the steepest curve first and the shallowest last. que: quercetin; kae: kaempferol; fis: fisetin; gen: genistein; dai: daidzein; nar: naringenin; hes: hesperetin; lut: luteolin; api: apigenin; asc-a: ascorbic acid, a-toc: *α*-tocopherol. ^∗^*p* < 0.05 compared to trolox, ^∗∗∗^*p* < 0.005 compared to trolox.

Previous reports have found that flavonoids and vitamins showed antioxidant capacity in FRAP and ABTS assays [74]. Interestingly, in our ABTS and FRAP assays, the flavonoids quercetin and fisetin (and in the FRAP assay luteolin) showed higher values than the water-soluble vitamin E analogue trolox while apigenin, daidzein, and naringenin showed very low values (Figure 4). By correlating ABTS and FRAP values with the total number of hydroxy groups in the molecule as well as the number of hydroxy groups in the B ring (Table 1), our results reflect previous observations describing structure-activity relationships for radical scavenging polyphenols [75]. The presence of a catechol structure in the B ring could further explain why fisetin with 4 hydroxy groups is more potent in the ABTS and FRAP assays than kaempferol and ascorbic acid which also have 4 hydroxy groups.

By carrying out correlation analyses, we found a very strong correlation between FRAP and ABTS (*R* = 0.98, Table 1). However, while it seemed that FRAP and ABTS values correlated strongly (*R* = 0.73 and 0.59, resp.) with the total number of hydroxy groups in the molecule and even stronger with the number of hydroxy groups in the B ring (*R* = 0.82 and 0.73, resp.), the presence of hydroxy groups did not correlate with transcription factor-induced reporter gene activation (Table 1).

### 3.4. Pro- and Antioxidant Potential of Flavonoids

Paradoxically, flavonoids may protect from oxidative stress by acting as pro-oxidants. Although in cell-free in vitro assays they show radical scavenging and reducing activity (Figure 4), they are unlikely to exert a protective role towards antioxidative stress by scavenging radicals in vivo. Their low concentration inside the body and the slow reaction rate constants of such nonenzymatic (as compared to enzyme-catalysed) radical scavenging point to the notion that flavonoids stimulate endogenous (e.g., enzymatic) antioxidant defence [76–78]. Kelch-like ECH-associated protein 1 (Keap1) binds to Nrf2 in the cytosol which leads to proteasomal degradation of Nrf2 and prevents transcription of its target genes [79]. After oxidation to quinones, flavonoids could—either directly or via the formation of reactive oxygen species—cause dissociation of Nrf2 from its inhibitor Keap1, inducing nuclear translocation of the transcription factor, leading to expression of genes coding for antioxidant enzymes [77]. In our cell culture model reporting Nrf2-binding to ARE, flavonoids causing such a pro-oxidative, Nrf2-activating effect may have induced ARE-driven luciferase expression. The “xenohormesis hypothesis” postulates that induction of stress signalling pathways by subtoxic doses of a stressor such as a dietary flavonoid may prepare the organism to better defend itself from stress arising during ageing and thus extend lifespan [80].

## 4. Conclusion

While free radical scavenging properties of flavonoids do not correlate with the capability of these secondary plant metabolites to induce longevity-associated transcription factors, more in vivo research is needed to understand how flavonoids may benefit longevity. Because of the possibly interconnected signalling pathways that are induced and the impact of metabolism on the bioavailability of these compounds, further research in more complex models is desirable.

## Supplementary Material

Supplemental Table 1: Table showing the antioxidant capacity relative to trolox (slope of the flavonoid / vitamin curve divided by the slope of the trolox curve) in the ABTS assay and the FRAP assay, the p-values from the statistics comparing the treatment with the compound to the treatment with the vehicle control in the luciferase assays and the number of hydroxy groups in the compound (further divided into hydroxyl groups in the A, B and C ring where applicable).

## Figures and Tables

**Table 1:** Correlation coefficients with *p* values. Significant correlations are printed in bold (*p* < 0.05) and correlations printed in italics show a trend (*p* < 0.1). We could not find any correlation between the antioxidant capacity as measured by the FRAP or ABTS assay and the luciferase assays. The total number of hydroxy groups in the molecule and especially the number of hydroxy groups in the flavonoid B-ring correlated strongly with the outcome in the FRAP and ABTS assays. Furthermore, ARE- and FHRE-activation correlated strongly with each other, and, albeit to a lesser extent, with PPAR*γ*-activation.

Pearson correlation coefficients (*p* values)	Total OH	OH in A ring	OH in B ring	OH in C ring	PPAR-UAS activation	FHRE activation	ARE activation	FRAP
ABTS	*0.59* (0.06)	−0.21 (0.59)	**0.73** (0.03)	0.52 (0.15)	0.05 (0.88)	0.09 (0.79)	0.45 (0.16)	**0.98** (<0.0001)
FRAP	**0.73** (0.01)	−0.21 (0.59)	**0.82** (0.001)	0.52 (0.15)	0.12 (0.72)	0.14 (0.70)	0.46 (0.16)	
ARE activation	0.43 (0.19)	−0.31 (0.42)	0.64 (0.06)	0.35 (0.36)	**0.64** (0.04)	**0.85** (0.001)		
FHRE activation	0.20 (0.56)	−0.31 (0.42)	0.37 (0.33)	−0.35 (0.36)	**0.67** (0.03)			
PPAR-UAS activation	0.26 (0.44)	−0.31 (0.42)	0.27 (0.48)	0.17 (0.66)				

## References

[1] Hertog M. G., Feskens E. J., Kromhout D. (1997). Antioxidant flavonols and coronary heart disease risk. *Lancet*.

[2] Hertog M. G., Kromhout D., Aravanis C. (1995). Flavonoid intake and long-term risk of coronary heart disease and cancer in the seven countries study. *Archives of Internal Medicine*.

[3] Pallauf K., Duckstein N., Rimbach G. (2016). A literature review of flavonoids and lifespan in model organisms. *The Proceedings of the Nutrition Society*.

[4] Williams R. J., Spencer J. P., Rice-Evans C. (2004). Flavonoids: antioxidants or signalling molecules?. *Free Radical Biology & Medicine*.

[5] Alam J., Stewart D., Touchard C., Boinapally S., Choi A. M., Cook J. L. (1999). Nrf2, a Cap'n'Collar transcription factor, regulates induction of the heme oxygenase-1 gene. *The Journal of Biological Chemistry*.

[6] Venugopal R., Jaiswal A. K. (1996). Nrf1 and Nrf2 positively and c-Fos and Fra1 negatively regulate the human antioxidant response element-mediated expression of NAD(P)H:quinone oxidoreductase1 gene. *Proceedings of the National Academy of Sciences of the United States of America*.

[7] Chorley B. N., Campbell M. R., Wang X. (2012). Identification of novel NRF2-regulated genes by ChIP-Seq: influence on retinoid X receptor alpha. *Nucleic Acids Research*.

[8] Brunet A., Bonni A., Zigmond M. J. (1999). Akt promotes cell survival by phosphorylating and inhibiting a forkhead transcription factor. *Cell*.

[9] Eijkelenboom A., Burgering B. M. (2013). FOXOs: signalling integrators for homeostasis maintenance. *Nature Reviews. Molecular Cell Biology*.

[10] Flachsbart F., Caliebe A., Kleindorp R. (2009). Association of FOXO3A variation with human longevity confirmed in German centenarians. *Proceedings of the National Academy of Sciences of the United States of America*.

[11] Moseti D., Regassa A., Kim W. K. (2016). Molecular regulation of adipogenesis and potential anti-adipogenic bioactive molecules. *International Journal of Molecular Sciences*.

[12] Scazzocchio B., Vari R., Filesi C. (2011). Cyanidin-3-O-beta-glucoside and protocatechuic acid exert insulin-like effects by upregulating PPARgamma activity in human omental adipocytes. *Diabetes*.

[13] Polvani S., Tarocchi M., Galli A. (2012). PPARgamma and oxidative stress: con(beta) catenating NRF2 and FOXO. *PPAR Research*.

[14] Leonardo C. C., Dore S. (2011). Dietary flavonoids are neuroprotective through Nrf2-coordinated induction of endogenous cytoprotective proteins. *Nutritional Neuroscience*.

[15] Medjakovic S., Mueller M., Jungbauer A. (2010). Potential health-modulating effects of isoflavones and metabolites via activation of PPAR and AhR. *Nutrients*.

[16] Huang C. Y., Chan C. Y., Chou I. T., Lien C. H., Hung H. C., Lee M. F. (2013). Quercetin induces growth arrest through activation of FOXO1 transcription factor in EGFR-overexpressing oral cancer cells. *The Journal of Nutritional Biochemistry*.

[17] Liu X., Yu Z., Huang X. (2016). Peroxisome proliferator-activated receptor gamma (PPARgamma) mediates the protective effect of quercetin against myocardial ischemia-reperfusion injury via suppressing the NF-kappaB pathway. *American Journal of Translational Research*.

[18] Arredondo F., Echeverry C., Abin-Carriquiry J. A. (2010). After cellular internalization, quercetin causes Nrf2 nuclear translocation, increases glutathione levels, and prevents neuronal death against an oxidative insult. *Free Radical Biology & Medicine*.

[19] Shukla S., Bhaskaran N., Babcook M. A., Fu P., Maclennan G. T., Gupta S. (2014). Apigenin inhibits prostate cancer progression in TRAMP mice via targeting PI3K/Akt/FoxO pathway. *Carcinogenesis*.

[20] Buchter C., Havermann S., Koch K., Watjen W. (2016). Isoxanthohumol, a constituent of hop (*Humulus lupulus* L.), increases stress resistance in *Caenorhabditis elegans* dependent on the transcription factor DAF-16. *European Journal of Nutrition*.

[21] Havermann S., Rohrig R., Chovolou Y., Humpf H. U., Watjen W. (2013). Molecular effects of baicalein in Hct116 cells and *Caenorhabditis elegans*: activation of the Nrf2 signaling pathway and prolongation of lifespan. *Journal of Agricultural and Food Chemistry*.

[22] Wruck C. J., Claussen M., Fuhrmann G. (2007). Luteolin protects rat PC12 and C6 cells against MPP+ induced toxicity via an ERK dependent Keap1-Nrf2-ARE pathway. *Journal of Neural Transmission Supplementum*.

[23] Eckers A., Sauerbier E., Anwar-Mohamed A. (2011). Detection of a functional xenobiotic response element in a widely employed FoxO-responsive reporter construct. *Archives of Biochemistry and Biophysics*.

[24] Brand A. H., Perrimon N. (1993). Targeted gene expression as a means of altering cell fates and generating dominant phenotypes. *Development*.

[25] Mukherjee R., Jow L., Croston G. E., Paterniti J. R. (1997). Identification, characterization, and tissue distribution of human peroxisome proliferator-activated receptor (PPAR) isoforms PPARgamma2 versus PPARgamma1 and activation with retinoid X receptor agonists and antagonists. *The Journal of Biological Chemistry*.

[26] Christensen K. B., Minet A., Svenstrup H. (2009). Identification of plant extracts with potential antidiabetic properties: effect on human peroxisome proliferator-activated receptor (PPAR), adipocyte differentiation and insulin-stimulated glucose uptake. *Phytotherapy Research: PTR*.

[27] Schrader E., Wein S., Kristiansen K., Christensen L. P., Rimbach G., Wolffram S. (2012). Plant extracts of winter savory, purple coneflower, buckwheat and black elder activate PPAR-gamma in COS-1 cells but do not lower blood glucose in Db/db mice in vivo. *Plant Foods for Human Nutrition*.

[28] Borenfreund E., Puerner J. A. (1985). Toxicity determined in vitro by morphological alterations and neutral red absorption. *Toxicology Letters*.

[29] Valacchi G., Rimbach G., Saliou C., Weber S. U., Packer L. (2001). Effect of benzoyl peroxide on antioxidant status, NF-kappaB activity and interleukin-1 alpha gene expression in human keratinocytes. *Toxicology*.

[30] Re R., Pellegrini N., Proteggente A., Pannala A., Yang M., Rice-Evans C. (1999). Antioxidant activity applying an improved ABTS radical cation decolorization assay. *Free Radical Biology & Medicine*.

[31] Benzie I. F., Strain J. J. (1996). The ferric reducing ability of plasma (FRAP) as a measure of “antioxidant power”: the FRAP assay. *Analytical Biochemistry*.

[32] R-Core-Team T. (2015). *A Language and Environment for Statistical Computing*.

[33] Verbeke G., Geert M. (2000). *Linear Mixed Models for Longitudinal Data*.

[34] Laird N. M., Ware J. H. (1982). Random-effects models for longitudinal data. *Biometrics*.

[35] Nakagawa S., Schielzeth H. (2013). A general and simple method for obtaining R2 from generalized linear mixed-effects models. *Methods in Ecology and Evolution*.

[36] Bretz F., Hothorn T., Westfall P. (2010). *Multiple Comparisons Using R*.

[37] Pallauf K., Bendall J. K., Scheiermann C. (2013). Vitamin C and lifespan in model organisms. *Food and Chemical Toxicology*.

[38] Ernst I. M., Pallauf K., Bendall J. K. (2013). Vitamin E supplementation and lifespan in model organisms. *Ageing Research Reviews*.

[39] Wang L., Waltenberger B., Pferschy-Wenzig E. M. (2014). Natural product agonists of peroxisome proliferator-activated receptor gamma (PPARgamma): a review. *Biochemical Pharmacology*.

[40] Bumke-Vogt C., Osterhoff M. A., Borchert A. (2014). The flavones apigenin and luteolin induce FOXO1 translocation but inhibit gluconeogenic and lipogenic gene expression in human cells. *PloS One*.

[41] Lee S. E., Jeong S. I., Yang H., Park C. S., Jin Y. H., Park Y. S. (2011). Fisetin induces Nrf2-mediated HO-1 expression through PKC-delta and p38 in human umbilical vein endothelial cells. *Journal of Cellular Biochemistry*.

[42] Saw C. L., Guo Y., Yang A. Y. (2014). The berry constituents quercetin, kaempferol, and pterostilbene synergistically attenuate reactive oxygen species: involvement of the Nrf2-ARE signaling pathway. *Food and Chemical Toxicology*.

[43] Paredes-Gonzalez X., Fuentes F., Jeffery S. (2015). Induction of NRF2-mediated gene expression by dietary phytochemical flavones apigenin and luteolin. *Biopharmaceutics & Drug Disposition*.

[44] Youns M., Abdel Halim Hegazy W. (2017). The natural flavonoid fisetin inhibits cellular proliferation of hepatic, colorectal, and pancreatic cancer cells through modulation of multiple signaling pathways. *PloS One*.

[45] Meeran S. M., Katiyar S. K. (2008). Cell cycle control as a basis for cancer chemoprevention through dietary agents. *Frontiers in Bioscience*.

[46] Bartholome A., Kampkotter A., Tanner S., Sies H., Klotz L. O. (2010). Epigallocatechin gallate-induced modulation of FoxO signaling in mammalian cells and *C. elegans*: FoxO stimulation is masked via PI3K/Akt activation by hydrogen peroxide formed in cell culture. *Archives of Biochemistry and Biophysics*.

[47] Perez-Cano F. J., Castell M. (2016). Flavonoids, inflammation and immune system. *Nutrients*.

[48] Ying B., Yang T., Song X. (2009). Quercetin inhibits IL-1 beta-induced ICAM-1 expression in pulmonary epithelial cell line A549 through the MAPK pathways. *Molecular Biology Reports*.

[49] Boesch-Saadatmandi C., Loboda A., Wagner A. E. (2011). Effect of quercetin and its metabolites isorhamnetin and quercetin-3-glucuronide on inflammatory gene expression: role of miR-155. *The Journal of Nutritional Biochemistry*.

[50] Kampkoetter A., Nkwonkam C. G., Zurawski R. F. (2007). Effects of the flavonoids kaempferol and fisetin on thermotolerance, oxidative stress and FoxO transcription factor DAF-16 in the model organism *Caenorhabditis elegans*. *Archives of Toxicology*.

[51] Grunz G., Haas K., Soukup S. (2012). Structural features and bioavailability of four flavonoids and their implications for lifespan-extending and antioxidant actions in *C. elegans*. *Mechanisms of Ageing and Development*.

[52] Willcox B. J., Donlon T. A., He Q. (2008). FOXO3A genotype is strongly associated with human longevity. *Proceedings of the National Academy of Sciences of the United States of America*.

[53] Buchter C., Ackermann D., Havermann S. (2013). Myricetin-mediated lifespan extension in *Caenorhabditis elegans* is modulated by DAF-16. *International Journal of Molecular Sciences*.

[54] Hertog M. G., Feskens E. J., Hollman P. C., Katan M. B., Kromhout D. (1993). Dietary antioxidant flavonoids and risk of coronary heart disease: the Zutphen Elderly Study. *Lancet*.

[55] Pallauf K., Giller K., Huebbe P., Rimbach G. (2013). Nutrition and healthy ageing: calorie restriction or polyphenol-rich “MediterrAsian” diet?. *Oxidative Medicine and Cellular Longevity*.

[56] Walle T. (2004). Absorption and metabolism of flavonoids. *Free Radical Biology & Medicine*.

[57] Arts M. J., Haenen G. R., Wilms L. C. (2002). Interactions between flavonoids and proteins: effect on the total antioxidant capacity. *Journal of Agricultural and Food Chemistry*.

[58] Hallenborg P., Petersen R. K., Kouskoumvekaki I., Newman J. W., Madsen L., Kristiansen K. (2016). The elusive endogenous adipogenic PPARgamma agonists: lining up the suspects. *Progress in Lipid Research*.

[59] Lehmann J. M., Moore L. B., Smith-Oliver T. A., Wilkison W. O., Willson T. M., Kliewer S. A. (1995). An antidiabetic thiazolidinedione is a high affinity ligand for peroxisome proliferator-activated receptor *γ* (PPAR*γ*). *The Journal of Biological Chemistry*.

[60] Nissen S. E., Wolski K. (2007). Effect of rosiglitazone on the risk of myocardial infarction and death from cardiovascular causes. *The New England Journal of Medicine*.

[61] Fang X. K., Gao J., Zhu D. N. (2008). Kaempferol and quercetin isolated from *Euonymus alatus* improve glucose uptake of 3T3-L1 cells without adipogenesis activity. *Life Sciences*.

[62] Xu N., Zhang L., Dong J. (2014). Low-dose diet supplement of a natural flavonoid, luteolin, ameliorates diet-induced obesity and insulin resistance in mice. *Molecular Nutrition & Food Research*.

[63] Feng X., Weng D., Zhou F. (2016). Activation of PPARgamma by a natural flavonoid modulator, apigenin ameliorates obesity-related inflammation via regulation of macrophage polarization. *eBioMedicine*.

[64] Shen P., Liu M. H., Ng T. Y., Chan Y. H., Yong E. L. (2006). Differential effects of isoflavones, from Astragalus membranaceus and Pueraria thomsonii, on the activation of PPARalpha, PPARgamma, and adipocyte differentiation in vitro. *The Journal of Nutrition*.

[65] Dang Z. C., Audinot V., Papapoulos S. E., Boutin J. A., Lowik C. W. (2003). Peroxisome proliferator-activated receptor gamma (PPARgamma) as a molecular target for the soy phytoestrogen genistein. *The Journal of Biological Chemistry*.

[66] Dowell P., Otto T. C., Adi S., Lane M. D. (2003). Convergence of peroxisome proliferator-activated receptor gamma and Foxo1 signaling pathways. *The Journal of Biological Chemistry*.

[67] Cho H. Y., Gladwell W., Wang X. (2010). Nrf2-regulated PPAR*γ* expression is critical to protection against acute lung injury in mice. *American Journal of Respiratory and Critical Care Medicine*.

[68] Kim J. J., Li P., Huntley J., Chang J. P., Arden K. C., Olefsky J. M. (2009). FoxO1 haploinsufficiency protects against high-fat diet-induced insulin resistance with enhanced peroxisome proliferator-activated receptor gamma activation in adipose tissue. *Diabetes*.

[69] Huang J., Tabbi-Anneni I., Gunda V., Wang L. (2010). Transcription factor Nrf2 regulates SHP and lipogenic gene expression in hepatic lipid metabolism. *American Journal of Physiology Gastrointestinal and Liver Physiology*.

[70] Pi J., Leung L., Xue P. (2010). Deficiency in the nuclear factor E2-related factor-2 transcription factor results in impaired adipogenesis and protects against diet-induced obesity. *The Journal of Biological Chemistry*.

[71] Nolte R. T., Wisely G. B., Westin S. (1998). Ligand binding and co-activator assembly of the peroxisome proliferator-activated receptor-gamma. *Nature*.

[72] Zhang T., Wang F., Xu H. X. (2013). Activation of nuclear factor erythroid 2-related factor 2 and PPARgamma plays a role in the genistein-mediated attenuation of oxidative stress-induced endothelial cell injury. *The British Journal of Nutrition*.

[73] Huang H. C., Nguyen T., Pickett C. B. (2000). Regulation of the antioxidant response element by protein kinase C-mediated phosphorylation of NF-E2-related factor 2. *Proceedings of the National Academy of Sciences of the United States of America*.

[74] Csepregi K., Neugart S., Schreiner M., Hideg E. (2016). Comparative evaluation of total antioxidant capacities of plant polyphenols. *Molecules (Basel, Switzerland)*.

[75] Amic D., Davidović-Amić D., Beslo D., Trinajstic N. (2003). Structure-radical scavenging activity relationships of flavonoids. *Croatica Chemica Acta*.

[76] Forman H. J., Davies K. J., Ursini F. (2014). How do nutritional antioxidants really work: nucleophilic tone and para-hormesis versus free radical scavenging in vivo. *Free Radical Biology & Medicine*.

[77] Tu T., Giblin D., Gross M. L. (2011). Structural determinant of chemical reactivity and potential health effects of quinones from natural products. *Chemical Research in Toxicology*.

[78] Croft K. D. (2016). Dietary polyphenols: antioxidants or not?. *Archives of Biochemistry and Biophysics*.

[79] Itoh K., Wakabayashi N., Katoh Y. (1999). Keap1 represses nuclear activation of antioxidant responsive elements by Nrf2 through binding to the amino-terminal Neh2 domain. *Genes & Development*.

[80] Lamming D. W., Wood J. G., Sinclair D. A. (2004). Small molecules that regulate lifespan: evidence for xenohormesis. *Molecular Microbiology*.

