# Supplementary material for: Flavonoids as Putative Inducers of the Transcription Factors Nrf2, FoxO, and PPARγ
Source: Oxid Med Cell Longev. 2017 Jul 6;2017:4397340. doi: 10.1155/2017/4397340 (PMC5518529; doi:10.1155/2017/4397340)
Supplement: Supplementary file 1 — Supplemental Table 1: Table showing the antioxidant capacity relative to trolox (slope of the flavonoid / vitamin curve divided by the slope of the trolox curve) in the ABTS assay and the FRAP assay, the p-values from the statistics comparing the treatment with the compound to the treatment with the vehicle control in the luciferase assays and the number of hydroxy groups in the compound (further divided into hydroxyl groups in the A, B and C ring where applicable). [file 4397340.f1.docx]

*Supplementary Data*

| substance | ABTS slope relative to trolox | FRAP slope relative to trolox | p-value ARE-activation | p-value FHRE-activation | p-value PPARɣ-UAS activation | n hydroxy  groups  (total, A:B:C) |
| --- | --- | --- | --- | --- | --- | --- |
| quercetin | 2.626  +/-0.277 | 2.815  +/-0.902 | <0.001 | <0.001 | <0.001 | 5, 2:2:1 C-ring desaturated |
| kaempferol | 0.347  +/-0.010 | 0.675  +/-0.504 | 0.001 | <0.001 | <0.001 | 4, 2:1:1 C-ring desaturated |
| fisetin | 3.092  +/-0.251 | 3.116  +/-0.299 | <0.001 | <0.001 | 0.279 | 4, 1:2:1 C-ring desaturated |
| genistein | 0.362  +/-0.160 | 0.099  +/-0.026 | 0.078 | 0.002 | 0.014 | 3, 2:1:0 C-ring desaturated |
| daidzein | 0.218  +/- 0.004 | 0.061  +/- 0.009 | <0.001 | <0.001 | <0.001 | 2, 1:1:0 C-ring desaturated |
| naringenin | 0.196  +/-0.024 | 0.060  +/-0.012 | 0.400 | 0.250 | 0.017 | 3, 2:1:0 C-ring saturated |
| hesperetin | 0.840  +/-0.055 | 0.736  +/-0.043 | 0.116 | 0.031 | 0.006 | 3, 2:1:0 C-ring saturated |
| luteolin | 0.667  +/- 0.172 | 1.182  +/- 0.254 | <0.001 | <0.001 | 0.001 | 4, 2:2:0 C-ring desaturated |
| apigenin | 0.052  +/-0.022 | 0.033  +/-0.011 | <0.001 | <0.001 | <0.001 | 3, 2:1:0 C-ring desaturated |
| ascorbic acid | 0.928  +/-0.140 | 1.006  +/-0.090 | 0.981 | 0.805 | 0.612 | 4 |
| α-tocopherol | 0.425  +/-0.174 | 0.430  +/-0.120 | 0.974 | 0.993 | 0.941 | 1 |

Supplemental Table 1: Table showing the antioxidant capacity relative to trolox (slope of the flavonoid / vitamin curve divided by the slope of the trolox curve) in the ABTS assay and the FRAP assay, the p-values from the statistics comparing the treatment with the compound to the treatment with the vehicle control in the luciferase assays and the number of hydroxy groups in the compound (further divided into hydroxyl groups in the A, B and C ring where applicable).
